# Gene and Biochemical Pathway Evaluation of Burns Injury via Protein-Protein Interaction Network Analysis

**DOI:** 10.31661/gmj.v8i0.1257

**Published:** 2019-08-07

**Authors:** Majid Rezaei-Tavirani, Vahid Mansouri, Mostafa Rezaei Tavirani, Mohammad Rostami-Nejad, Davood Bashash, Mona Zamanian Azodi

**Affiliations:** ^1^Faculty of Medicine, Iran University of Medical Sciences, Tehran, Iran; ^2^Proteomics Research Center, Faculty of Paramedical Sciences, Shahid Beheshti University of Medical Sciences, Tehran, Iran; ^3^Gastroenterology and Liver Diseases Research Center, Research Institute for Gastroenterology and Liver Diseases, Shahid Beheshti University of Medical Sciences, Tehran, Iran

**Keywords:** Burn, Gene, Biomarker, Protein-Protein Interaction Network, Biochemical Pathway

## Abstract

**Background:**

Severe burns injury can affect several vital systems in the body and can cause inflammation in organs such as the heart, liver, and kidney. Many inflammatory mediators and regulatory hormones related to burn injuries are recognized. In this study, the genes related to burn injury interacted via network analysis, and the central nodes were enriched through gene ontology (GO).

**Materials and Methods:**

Disease query of STRING database was used for data gathering, and the network was constructed using Cytoscape software version 3.6.0. After gene screening, the central nodes were enriched via GO analysis by ClueGO. The highlighted genes and pathways were clustered and analyzed in detail.

**Results:**

Among 1067 genes, 35 critical genes that are involved in the 14 highlighted biochemical pathways were recognized. Interpretation of the finding indicates that a number of central genes can be considered as potential biomarkers related to burn injury.

**Conclusion:**

Can we revise to "Burn injuries have features that are common to several diseases and increases their risk.

## Introduction

The incidence of burn injuries in developed countries has decreased, but burns are still considered the commonest form of injury and contribute to a considerable part of trauma cases in hospital emergencies [1]. Burns covering more than 30% of total body surface area (TBSA) can lead to burn shock. Release of inflammatory mediators coupled with considerable hypovolemia is observed in burn shock [2, 3]. Massive energy needs are found in burn patients. This hypermetabolic response affects cellular, tissue, and organ functions; the immune system; membrane transport; and organs such as the skin, heart, liver, and skeletal muscles, and it may also lead to a risk of infections [4, 5]. Release of stress hormones and inflammatory mediators and change in the regulation of cellular functions such as apoptosis and membrane transport are accompanied with gross alteration in the body physiology of burn patients [1]. Proteomics can provide a large number of proteins involved in diseases. In a proteomic study, 110 significant expression-changed proteins were identified in the plasma of burn patients. The main introduced proteins were involved in inflammatory and hypermetabolic response in burn victims [6]. In addition, 3250 significant expression-changed genes related to burn patients (false discovery rate <0.001 and fold change >2) are reported [7]. Vast molecular results about diseases can provide a new perspective around treatment, diagnosis, and prevention in medicine. However, it is essential to select the most important genes or proteins that play a crucial role in the incidence and development of diseases. System biology is a useful tool regarding the management of data. Graph theory is applied to organize large amounts of data as nodes in an interacted unit (a network) by connections (edges) [8-10]. The crucial nodes in the network are the genes or proteins that make more connections (known as hub nodes) or connect to the other nodes via more shortest paths (called bottleneck nodes) [11]. Related biochemical pathways of a certain gene set can be identified by gene ontology (GO) analysis. In this regard, the significant pathways can be selected and classified in the clusters [12]. In this study, the burn injury–related genes were analyzed via protein-protein interaction (PPI) network to introduce the central genes and their related biochemical pathways.

## Materials and Methods

The genes related to burn were obtained via disease query of STRING database. The PPI network was constructed using the Cytoscape software version 3.6.0 [9]. Confidence of 0.4 and undirected links were considered. The network was analyzed using the network analyzer application of the Cytoscape software. The main connected component was selected for further analysis. The network was visualized and the high-value degree nodes (degree above mean +2 SD) were identified as hub nodes. The hub nodes were categorized based on similar biological and pathological roles. The hub nodes were also enriched via GO by the CleuGO application of the Cytoscape software to determine related biochemical pathways. The pathways based on P-value <0.05 were selected and clustered. The significant pathways were classified based on similar biological and pathological characterizations.

## Results

**Figure 1:**
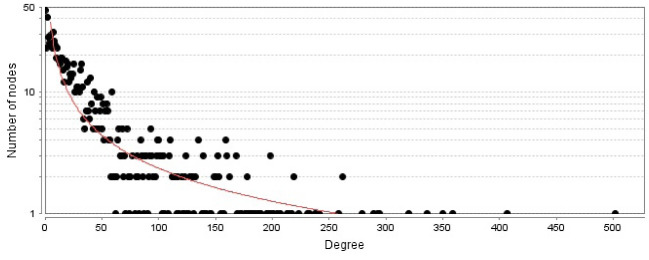
Degree distribution of nodes of burn PPI network is presented. Power law was fitted by y=ax^b^, a=150.19, b=-0.903, R-squared=0.789, and correlation=0.683.

**Figure 2:**
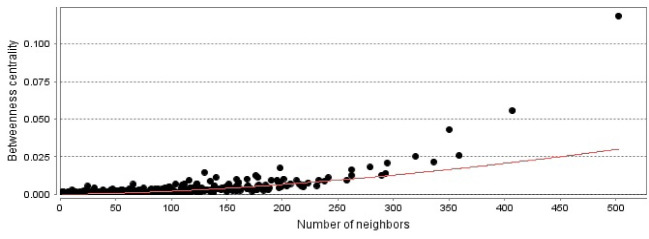
Betweenness distribution of nodes of burn PPI network is illustrated. Power law was fitted by y=ax^b^, a=000, b=1.654, R-squared=0.694, and correlation=0.841.

**Figure 3:**
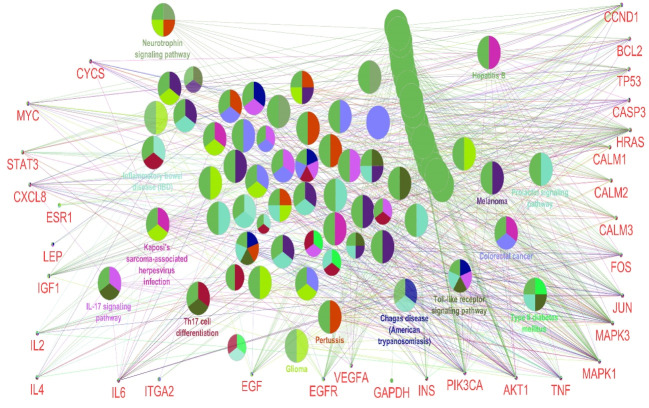
The hub-genes (that are arranged in the around of the network and are identified by red color) of burn PPI network and their related biochemical pathways (the circles) which were recognized by ClueGO are shown. The each colored part of a circle refers to a certain pathway for example the darkest red color corresponds to Th17 cell differentiation while the darkest blue color refers to Melanoma. If a circle is included the darkest red and blue colors talk about Th17 cell differentiation and Melanoma pathways. The name of circle is determined based of les P-value amount of pathways.

The PPI network including a main connected component and 47 isolated nodes was constructed and analyzed. The main connected component was characterized by 1020 nodes, 23,425 edges, and density of 0.041. No self-loops or multi-edge node pairs were detected. Degree distribution (Figure-1) was fitted by a power law that refers to scale-free network properties. Betweenness distribution was fitted to a power law (Figure-2). Central nodes such as hub nodes and bottleneck nodes are critical nodes that play crucial roles in the integrity of a network. 35 hub nodes as central genes were identified for burn network and are tabulated in Table-1. As interpretation of the roles of the 35 central nodes needs extensive details, the nodes based on functional roles were classified into six groups (Table-2). However, there is an overlap between the introduced groups. On the other hand, the 35 central genes were enriched by GO analysis, and 14 related biochemical pathways were identified (Figure-3). Similar to the central nodes, the resulted biochemical pathways were categorized into six classes of pathways and are shown in Table-3.

## Discussion

As mentioned in the Results section, a large number of genes (more than 1000) are involved in the constructed network. As the density of the network that corresponds to the frequency of connections between the nodes is considered to be between zero and one, very low amount of network density (0.041) refers to poor connections between the nodes of the burn network. This finding may have resulted from the unknown properties of the determined related genes in the literature. In the scale-free networks, there are limited nodes that are characterized by a large number of connections versus the large number of the nodes with poor links. The results indicate that the burn PPI network is a scale-free network. As described in the Methods section, the cut-off value for the degree was calculated to be 168, which discriminates the nodes with a high-value degree from the other genes. This finding is reflected in Figure-2, where the nodes deviate from the power law. For better understanding, the determined central nodes are classified. The first class includes only albumin. Albumin is a critical protein in the body and is responsible for a vast variety of vital roles in body maintenance. Transport of several hormones, drugs, metabolites, and other substances and its crucial role in the regulation of osmotic pressure are the well-known functions of albumin. The various opinions about usage of albumin in the resuscitation of patients with burn injury are discussed in several studies [13 and 14]. The second class consists of metabolic and energy expenditure genes, which include insulin (*INS*), cytochrome C (*CYCS*), 2, 4-dienoyl CoA reductase-1 (*DECR1*), glyceraldehyde-3-phosphate dehydrogenase (*GAPDH*), and leptin (*LEP*). However, the members of this class may play other important roles in the body. *INS* as a glucose level regulator; *CYCS* as one of the electron transfer elements; *DECR1* as an enzyme involved in beta-oxidation, which plays a key role in the degradation of fatty acids; *GAPDH* with a metabolic role and diverse cellular functions; and *LEP* that regulates energy expenditure by the inhibition of hunger play crucial roles in the metabolism and energy production [15-19]. Metabolic changes in patients with burns are well-known processes that have been studied several years ago [20].

As it is reflected in the name of the members of class 3 genes, they are oncogenes or cancer-related genes [21-23]. Reduced Akt/PKB kinase activity after a burn injury is reported by Lu *et al.* [24], whereas an enhanced amount of TNFα after a burn injury is reported by Luo *et al.* [25]. On the other hand, there are common regulatory effects between the introduced class 3 genes. For example, regulation of TP53 expression by Akt/PIK3CA is discussed by McCubrey *et al.* [26]. The role of several interleukins such as IL-1, IL-2, IL-6, and IL-12 in burn injury patients is investigated. It has been reported that the level of IL-6 in patients with burn injury was increased, and it reached a maximum value after a week [27-30]. It is reported that EGF enhances wound healing in patients with burn injury [31]. An investigation showed that calmodulin level in the burn blister fluid is three times greater than that found in the plasma [32, 33]. It is considered as a member of class 5 of central genes. There is evidence that BCL2 level in enterocytes is decreased by a severe oxidative stress reaction in 30% TBSA burn rats [34, 35]. Recently Aykac *et al.*. confirmed that expression of TNFα, IL-1β, CASP3, and CASP9 in burn injury rat models is changed [36, 37]. Amyloid beta (A4) precursor protein, coagulation factor II (thrombin), and estrogen receptor 1 are involved mostly in Alzheimer disease, blood coagulation, and reproduction [38-40]. However, they are also involved in cancer and other diseases that are related to the mentioned genes [40]. The findings of Figure-3 and Table-3 are confirmed by the pathways related to the central nodes that are discussed here. Class 1 pathways in Table-3 can be related to the class 4 central nodes in Table-2. Both the classes reveal immunological features of burn. Group 2 of central nodes and biochemical pathways refer to the metabolic alteration in burn patients. Elements of class 3 in both Tables-2 and 3 represent common critical points between cancer and burn. As most part of the infection response in the body, similar to the burn response, is involved with the immune system [41, 42], there is a correlation between both group 4 of pathways and central nodes. The pathway groups 5 and 6 and class 6 of central nodes signify the common aspects of burn with other diseases such as reproductive and neurological disorders. The vast variety of genes involved in burn injuries reflects its common roots with different diseases. Severe immunological response, gross metabolic alteration, regulation changes of cell function, neurodegenerative disorders, reproductive diseases, and even cancer are highlighted as possible secondary diseases that may pose as a risk to the patients with burn injuries.

## Conclusion

The highlighted central nodes and biochemical pathways related to burn indicate the common features of this trauma with the different types of diseases. It is possible that the quantitative evaluation of these crucial genes in patients with burn injuries reduces the risk of certain correlated diseases.

## Acknowledgment

This project is supported by Shahid Beheshti University of Medical Sciences (grant number: 25804).

## Conflict of interest

There is no conflict of interest.

**Table 1:** Hub-nodes of Burn PPI Network are shown. **D** and** BC** Refer to Degree and Betweenness Centrality Respectively

**R**	** name**	**description**	**D**	**BC**
**1**	ALB	albumin	502	0.12
**2**	INS	insulin	407	0.06
**3**	GAPDH	glyceraldehyde-3-phosphate dehydrogenase	359	0.03
**4**	TP53	tumor protein p53	350	0.04
**5**	IL6	interleukin 6 (interferon, beta 2)	336	0.02
**6**	AKT1	v-akt murine thymoma viral oncogene homolog 1	320	0.03
**7**	EGF	epidermal growth factor	294	0.01
**8**	TNF	tumor necrosis factor	293	0.02
**9**	VEGFA	vascular endothelial growth factor A	289	0.01
**10**	JUN	jun proto-oncogene	279	0.02
**11**	EGFR	epidermal growth factor receptor	262	0.02
**12**	PIK3CA	phosphatidylinositol-4,5-bisphosphate 3-kinase, catalytic subunit alpha	262	0.01
**13**	IL8	interleukin 8	258	0.01
**14**	FOS	FBJ murine osteosarcoma viral oncogene homolog	241	0.01
**15**	MYC	v-myc myelocytomatosis viral oncogene homolog (avian)	238	0.01
**16**	MAPK1	mitogen-activated protein kinase 1	233	0.01
**17**	MAPK3	mitogen-activated protein kinase 3	231	0.01
**18**	IGF1	insulin-like growth factor 1 (somatomedin C)	223	0.01
**19**	BCL2	B-cell CLL/lymphoma 2	219	0.01
**20**	F2	coagulation factor II (thrombin)	215	0.01
**21**	IL2	interleukin 2	213	0.01
**22**	IL4	interleukin 4	207	0.01
**23**	CALM1	calmodulin 1 (phosphorylase kinase, delta)	201	0.01
**24**	DECR1	2,4-dienoyl CoA reductase 1, mitochondrial	198	0.01
**25**	STAT3	signal transducer and activator of transcription 3 (acute-phase response factor)	198	0.02
**26**	HRAS	v-Ha-ras Harvey rat sarcoma viral oncogene homolog	198	0.01
**27**	ESR1	estrogen receptor 1	196	0.01
**28**	CALM2	calmodulin 2 (phosphorylase kinase, delta)	195	0.01
**29**	CALM3	calmodulin 3 (phosphorylase kinase, delta)	190	0.01
**30**	CASP3	caspase 3, apoptosis-related cysteine peptidase	185	0.01
**31**	LEP	leptin	179	0.01
**32**	APP	amyloid beta (A4) precursor protein	178	0.01
**33**	ITGA2	integrin, alpha 2 (CD49B, alpha 2 subunit of VLA-2 receptor)	176	0.01
**34**	CCND1	cyclin D1	169	0.01
**35**	CYCS	cytochrome c, somatic	168	0.01

**Table 2:** Classified hub-nodes of burn PPI Network are presented. The name of group is determined based on roles of genes in body. For example the genes of group-3 are known as cancer marker as it is mentioned in the related references.

**R**	** Genes**	**Group name**	**Referrences**
**1**	ALB	albumin	
**2**	INS, CYCS, DECR1, GAPDH, LEP	Metabolic and energy expenditure genes	(13-17)
**3**	TP53, AKT1, TNF, JUN, FOS, MYC, HRAS, PIK3CA, CCND1, ITGA2	Cancer markers	(18-20)
**4**	IL2, IL4, IL6, IL8, EGF, EGFR, VEGFA, IGF, STAT3	Interleukins and growth factors	(21, 22)
**5**	MAPK1, MAPK3, CALM1,CALM2, CALM3, BCL2, CASP3	Cell function regulators	(23-26)
**6**	APP, F2, ESR1	Other genes	

**Table 3:** Classified related pathways of burn PPI network are shown Group name was determined based on the common features pathways. For example the first group are affected by immunological events in body so are marked as Immunological related pathways.

**R**	** Pathways**	**Group name**
1	Inflammatory bowel disease (IBD), IL-17 signaling pathway, Toll-like receptor signaling pathway, Th17 cell differentiation	Immunological related pathways
2	Type II diabetes mellitus	Metabolic related pathway
3	Glioma, Colorectal cancer, Melanoma	Cancer related pathways
4	Kaposi,s sarcoma-associated herpesvirus infection, Chagas disease (American trypanosomiasis), Hepatitis B, Pertussis (bacteri)	Infection related pathways
5	Neurotropin signaling pathway	Nervous system related pathway
6	Prolactin signaling pathway	Reproduction and lactation related pathway
